# Proteomic and metabolomic profiling of plasma uncovers immune responses in patients with Long COVID-19

**DOI:** 10.3389/fmicb.2024.1470193

**Published:** 2024-12-27

**Authors:** Yulin Wei, Hongyan Gu, Jun Ma, Xiaojuan Mao, Bing Wang, Weiyan Wu, Shiming Yu, Jinyuan Wang, Huan Zhao, Yanbin He

**Affiliations:** ^1^Department of Pulmonary and Critical Care Medicine, Affiliated Nantong Hospital of Shanghai University (The Sixth People’s Hospital of Nantong), Nantong, Jiangsu, China; ^2^Key Laboratory of Digital Technology in Medical Diagnostics of Zhejiang Province, Hangzhou, China; ^3^Dian Diagnostics Group Co., Ltd., Hangzhou, Zhejiang, China

**Keywords:** Long COVID, mechanism, proteomics, metabolomics, plasma

## Abstract

Long COVID is an often-debilitating condition with severe, multisystem symptoms that can persist for weeks or months and increase the risk of various diseases. Currently, there is a lack of diagnostic tools for Long COVID in clinical practice. Therefore, this study utilizes plasma proteomics and metabolomics technologies to understand the molecular profile and pathophysiological mechanisms of Long COVID, providing clinical evidence for the development of potential biomarkers. This study included three age- and gender-matched cohorts: healthy controls (*n* = 18), COVID-19 recovered patients (*n* = 17), and Long COVID patients (*n* = 15). The proteomics results revealed significant differences in proteins between Long COVID-19 patients and COVID-19 recovered patients, with dysregulation mainly focused on pathways such as coagulation, platelets, complement cascade reactions, GPCR cell signal transduction, and substance transport, which can participate in regulating immune responses, inflammation, and tissue vascular repair. Metabolomics results showed that Long COVID patients and COVID-19 recovered patients have similar metabolic disorders, mainly involving dysregulation in lipid metabolites and fatty acid metabolism, such as glycerophospholipids, sphingolipid metabolism, and arachidonic acid metabolism processes. In summary, our study results indicate significant protein dysregulation and metabolic abnormalities in the plasma of Long COVID patients, leading to coagulation dysfunction, impaired energy metabolism, and chronic immune dysregulation, which are more pronounced than in COVID-19 recovered patients.

## 1 Introduction

Post-acute sequelae of viral infection refer to the persistence of various degrees of physical, cognitive, and emotional impairments that may continue after recovery from an acute viral infection. Most people infected with COVID-19 fully recover, but existing evidence suggests that approximately 10–20% of individuals experience various mid- and long-term effects after recovering from the initial disease. These sequelae are referred to as “Long COVID” (Shah et al., 2021; Greenhalgh et al., 2020; Raveendran, 2021). The cumulative incidence of Long COVID is sixfold higher than similar viral infections (Lippi et al., 2023). According to conservative estimates, at least 76 million people globally may suffer from Long COVID (Davis et al., 2023).

Common symptoms associated with Long COVID-19 sequelae include fatigue, breathlessness, cognitive dysfunction (e.g., confusion, forgetfulness, lack of concentration, or mental fog), muscle spasms, cough, sleep disturbances, tachycardia, anxiety, chest pain and joint pain (Carfì et al., 2020; Huang et al., 2021; Asadi-Pooya et al., 2022). Long COVID patients may experience functional impairments in daily life, with over 200 symptoms identified that affect multiple organ systems (Lippi et al., 2023). Recent studies identified fatigue, cognitive impairment, joint pain, anxiety, and depression as the main clinical symptoms of long-term COVID-19 (Davis et al., 2021; Chen et al., 2022). Furthermore, Long COVID is also a risk factor for complications, with common comorbidities including cardiovascular, cerebrovascular diseases, chronic fatigue syndrome, and autonomic nervous system dysfunction (Munblit et al., 2022; Xie et al., 2022; Xie and Al-Aly, 2022; Mancini et al., 2021; Kedor et al., 2022). Due to the non-specific clinical presentations of Long COVID, lack of follow-up screenings, and absence of diagnostic biomarkers (Raveendran, 2021; Duerlund et al., 2022), there is a potential for delayed treatment and increased risk of complications in clinical settings. Therefore, it is crucial to explore the potential pathogenic mechanisms of the disease, which would provide clinical evidence for the development of specific diagnostic biomarkers for Long COVID and the optimization of precise clinical treatment strategies.

The pathogenesis of Long COVID is complex, and several hypotheses have been proposed regarding its mechanism, including persistent storage of SARS-CoV-2 in tissues (Proal and VanElzakker, 2021), immune dysregulation with or without reactivation of latent pathogens such as herpes viruses (Phetsouphanh et al., 2022), microvascular coagulation with endothelial dysfunction (Haffke et al., 2022), and dysfunction in neural signal transmission (Spudich and Nath, 2022). However, the potential pathogenic mechanisms of Long COVID have not been fully elucidated. Proteins are direct products of the genome, while metabolites are functional products of interactions between the host and the environment, disease states, clinical information, and other factors (He et al., 2022; Qu et al., 2023; Tian et al., 2022). Utilizing multi-omics can characterize the biological processes behind COVID-19. Hence, our goal is to study the metabolome and proteome to comprehensively understand the host response and identify potential molecular mechanisms and biomarkers associated with Long COVID-19.

## 2 Methods

### 2.1 Subjects and study design

**Figure 1:**
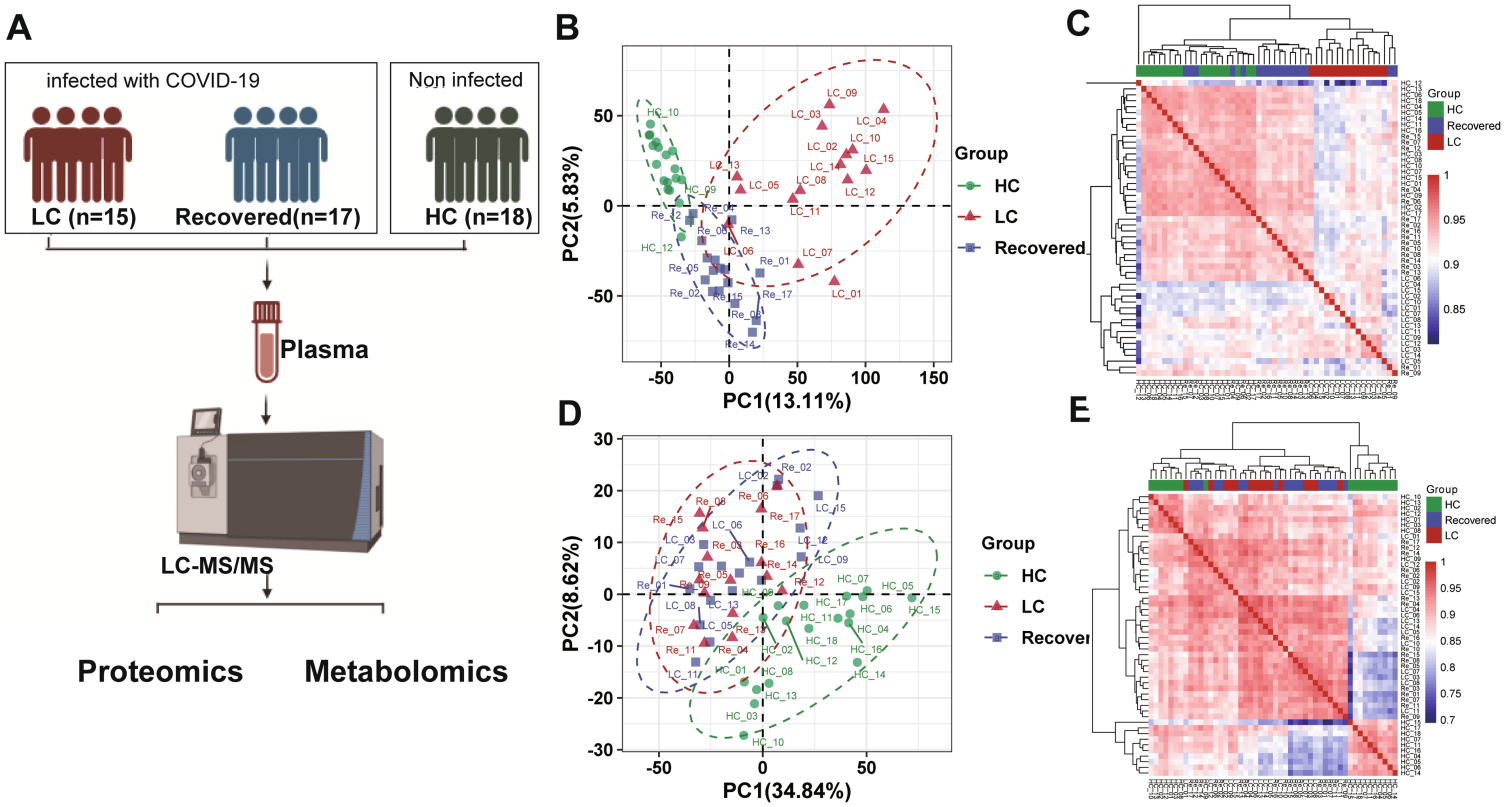
Proteomics and metabolomics analysis and profiling result. **(A)** Flowchart of study design. **(B)** PCA plot of proteomic analysis. **(C)** Proteomics heatmap of association analysis among three patient group. **(D)** PCA plot of metabolomics analysis. **(E)** Metabolomics heatmap of association analysis among three patient group.

All participants were recruited from the Sixth People’s Hospital of Nantong (Jiangsu, China). A total of 50 plasma samples were collected, including 18 from healthy individuals (HC group), 17 from recovered COVID-19 patients (Recovered group), and 15 from Long COVID patients. All enrolled patients met the diagnostic criteria, clinical classification, and discharge criteria outlined in the “Chinese Clinical Guidance for COVID-19 Pneumonia Diagnosis and Treatment (7th edition)” published by the China National Health Commission. Long COVID (LC group) was defined as the presence of one or more persistent COVID-19–related symptoms that could not be explained by an alternative diagnosis. While common Long COVID symptoms include smell and taste dysfunction, fatigue, shortness of breath, and cognitive dysfunction, over 200 different symptoms have been reported to impact daily functioning. The Recovered group comprised individuals who had been infected with COVID-19, recovered for more than 6 months, and currently had no symptoms associated with Long COVID. Additionally, 18 age-matched individuals without COVID-19 or lung abnormalities were recruited as healthy volunteers (Figure 1A). Written informed consent was obtained from both COVID-19 survivors and healthy controls after approval from the Research Ethics Commission of the hospital (NTLYLL2022049).

### 2.2 Sample collection

Venous blood was collected from participants and processed within 12 h to isolate plasma. And the plasma was obtained by centrifugation at 4°C (2000 rpm, 10 min). Then, all plasma samples were stored in a refrigerator at −80°C for proteomic and metabolomic analysis.

### 2.3 Plasma sample preparation for DIA analysis

Plasma samples were prepared in accordance with serum samples as described in our previous studies (Chen et al., 2021; Zhang et al., 2022). Plasma from each sample was mixed with the reaction solution buffer (1% sodium deoxycholate, 10 mM tris(2-carboxyethyl) phosphine hydrochloride, 40 mM 2-chloroacetamide). The reaction was carried out at 56°C for 30 min for protein denaturation, disulfide bond reduction, and cysteine SH alkylation. The sample was passed through a Sep-Pak C18 cartridge (1 cm3 50 mg, tC18 Cartridges, Waters, Milford, MA) conditioned and washed by 0.1% Formic acid (FA) solution for low-abundance proteins enrichment and then eluted by 75% acetonitrile in 0.1% FA (Capriotti et al., 2012; Lin et al., 2019). Then, each sample was diluted with an equal volume of H_2_O, and trypsin was added at a ratio of 1:50 (enzyme: protein, w/w) and incubated overnight at 37°C for digestion. After centrifugation (12,000 × g, 15 min), the supernatant was subjected to peptide purification using self-made desalting columns. The peptide eluate was vacuum drying process and spiked with iRT peptides before DIA analysis.

### 2.4 Liquid chromatography-mass spectrometry for proteome analysis

The peptides were dissolved in mobile phase A, which consisted of 2% acetonitrile and 0.1% formic acid. Following centrifugation at 20,000 × g for 10 min, the supernatant was separated using an UltiMate 3,000 UHPLC system (Thermo, United States). In brief, the peptides underwent enrichment in the trap column and then proceeded to the connected self-packed C18 column (1.8 μm, 150 μm × 350 cm) for separation at a flow rate of 500 nL/min. The elution of peptides utilized the following gradient: 0–5 min, 5% mobile phase B (98% acetonitrile, 0.1% formic acid); 5–90 min, 5–25% mobile phase B; 90–100 min, 25–35% mobile phase B; 100–108 min, 35–80% mobile phase B; 108–113 min, 80% mobile phase B; 113–120 min, 5% mobile phase B. The liquid-phase separated peptides were ionized using a nano ESI source and subsequently connected to a Q-Exactive HF tandem mass spectrometer (MS) (Thermo, United States). For the analysis of the plasma proteome in each subject, the Q Exactive HF instrument operated in the data-independent acquisition (DIA) mode, alternating between full-scan MS and MS/MS acquisition. The MS1 scan range was set at 400–1250 m/z with a resolution of 120,000 and an MIT of 50 ms. All precursor ions were directed to collision cells for fragmentation by high-energy collision dissociation (HCD). The MS/MS resolution was set at 30,000, the maximum fill time at automatic, and the AGC target at 1e6. DIA was conducted using a variable isolation window, with a total of 45 windows. During the proteomic analysis, we incorporated Quality Control (QC) samples throughout the detection process and subsequently assessed the stability among the QC samples by calculating the coefficient of variation (CV) of the proteins.

### 2.5 DIA data analysis

Identification and Quantification of proteins were generated with Spectronaut version 14.2 (Biognosys, https://biognosys.com/shop/spectronaut) (Bruderer et al., 2015) against a UNIPROT human database (only reviewed entries, 20,433 sequences) and the SARS-CoV-2 UNIPROT database. All the parameters were default. In order to ensure better alignment between different samples, we used iRT peptides to normalize the retention time alignment of different samples. The FDR was estimated using the mProphet scoring algorithm with 1% FDR control at the peptide-spectrum match, peptide, and protein levels. Proteins with missing ratios exceeding 80% in were excluded from the proteomics dataset. Missing values in the dataset were imputed with using the K-Nearest Neighbors (KNN) method (Jin et al., 2021). Next, the R package Msstats was used for log2 transformation, normalization, and *p* value calculation of the data (Choi et al., 2014). Differential expression protein screening needs to meet the following three conditions: (1) *p* value <0.05, (2) Fold change ≥1.5 or Fold change≤0.67, and (3) The pvalue were then adjusted using the Benjamini-Hochberg correction (qvalue <0.05).

### 2.6 Sample preparation for metabolome analysis

For comprehensive metabolite detection, both hydrophilic and hydrophobic metabolites were extracted and analyzed following a previously established method (Wu et al., 2020). To extract hydrophilic compounds, plasma samples were thawed on ice and vortexed for 10 s. The addition of six volumes of pure methanol to one volume of plasma samples was followed by thorough mixing for 3 min and centrifugation at 12,000 rpm for 10 min at 4°C. The resulting supernatant was collected and underwent another round of centrifugation at 12,000 rpm for 5 min at 4°C. The final supernatant was collected for LC–MS/MS analysis. To extract hydrophobic compounds, plasma samples were thawed on ice, vortexed for 10 s, and centrifuged at 3000 rpm for 5 min at 4°C. Subsequently, the plasma samples were thoroughly mixed with 1 mL of a lipid extract mixture (composed of methanol, tert-butyl methyl ether, and an internal standard mixture) for 15 min. This mixture was then combined with 200 μL of water, vortexed for 1 min, and centrifuged again at 12,000 rpm for 10 min at 4°C. The resulting supernatant was extracted, concentrated, dissolved in 200 μL of mobile phase B (consisting of acetonitrile/isopropanol at a ratio of 10%/90% (v/v), containing 0.04% acetic acid and 5 mM ammonium formate), and subjected to LC–MS/MS analysis.

### 2.7 Untargeted UPLC–MS/MS analysis

All UPLC–MS/MS analyses were conducted using the ACQUITY 2D UPLC system from Waters (Milford, MA, United States) and the Q Exactive HF hybrid Quadrupole-Orbitrap system from Thermo Fisher Scientific (San Jose, United States). The mass spectrometer operated at a resolution of 35,000 mass units at 200 m/z. In the first UPLC–MS/MS method, positive electrospray ionization (ESI) was employed with a C18 column (UPLC BEH C18, 2.1 × 100 mm, 1.7 μm; Waters). Mobile solutions composed of water and methanol containing 0.05% perfluorooctanoic acid (PFPA) and 0.1% formic acid (FA), with a final pH of 3, were used for gradient elution. The gradient elution, with the polar mobile phases ranging from 5 to 95%, was performed within a 7 min run. The second method also utilized the QE in positive ESI mode, with the same C18 column as in the first method. The mobile phase solutions for more hydrophobic compounds consisted of water, acetonitrile, methanol, 0.01% FA, and 0.05% PFPA at pH 3. For the third UPLC–MS/MS method, the QE was operated under negative ESI mode, and a C18 column was employed with mobile solutions containing methanol and water in 6.5 mM ammonium bicarbonate at pH 8. In the fourth method, an HILIC UPLC column (UPLC BEH Amide, 2.1 × 150 mm, 1.7 μm; Waters) was used, and the mobile solutions consisted of water and acetonitrile with 10 mM ammonium formate at pH 10.8. The gradient elution, reducing the polar mobile phase from 80 to 20%, occurred within a 7 min run. The QE was operated under negative ESI mode for this method as well. Analysis using the QE mass spectrometer involved alternating MS and data-dependent MS2 scans with dynamic exclusion. The scan range was set from 70 to 1,000 m/z, and the MS capillary temperature was 350°C, with a sheath gas flow rate of 40 and an auxiliary gas flow rate of 5 for both positive and negative methods.

The hydrophilic compounds were injected into a Waters ACQUITY UPLC HSS T3 column (1.8 μm, 2.1 mm × 100 mm). The column temperature, flow rate, and injection volume were set at 40°C, 0.4 mL/min, and 2 μL, respectively. The mobile phase comprised water (A) containing 0.1% formic acid and acetonitrile (B) containing 0.1% formic acid. The gradient elution involved an increase from 5% B to 90% B over 11 min, which was then maintained for 1 min before decreasing to 5% B over 2 min. Mass spectrometric scans were acquired using a 6,500+ QTRAP^®^ LC–MS/MS System equipped with an electrospray ionization (ESI) Turbo Ion-Spray interface. The system operated in both positive and negative ion modes and was controlled by Analyst 1.6.3 software (Sciex). The ESI source parameters were as follows: a source temperature of 500°C, an ion spray voltage of 5,500 V in positive ion mode (or − 4,500 V in negative ion mode), ion source gas I at 55 psi, ion source gas II at 60 psi, curtain gas at 25 psi, and collision-activated dissociation (CAD) set to high.

Meanwhile, the hydrophobic compounds were injected into a Waters AccucoreTM C30 column (2.6 μm, 2.1 mm × 100 mm). The column temperature, flow rate, and injection volume were set at 45°C, 0.35 mL/min, and 2 μL, respectively. The mobile phase consisted of acetonitrile/water (60%/40%, v/v) with 0.1% formic acid and 10 mmol/L ammonium formate (A), as well as acetonitrile/isopropanol (10%/90% v/v) with 0.1% formic acid and 10 mmol/L ammonium formate (B). The gradient elution involved an increase from 20% B to 95% B over 15.5 min. This composition was maintained for 2 min before decreasing to 20% B over 2.5 min. Mass spectrometric scans were acquired using a 6,500+ QTRAP^®^ LC–MS/MS System equipped with an ESI Turbo Ion-Spray interface. The system operated in both positive and negative ion modes and was controlled by Analyst 1.6.3 software (Sciex). The ESI source parameters were as follows: a source temperature of 500°C, an ion spray voltage of 5,500 V in positive ion mode (or − 4,500 V in negative ion mode), ion source gas I at 45 psi, ion source gas II at 55 psi, curtain gas at 35 psi, and collision-activated dissociation (CAD) set to medium.

Instrument tuning and mass calibration were performed with 10 and 100 μmol/L polypropylene glycol solutions in the triple quadrupole (QQQ) mode. Based on the self-built database and metabolite information in the public database, the materials were qualitatively analyzed according to the secondary spectrum information and the isotope signal was removed during the analysis. QQQ scans were acquired as multiple reaction monitoring (MRM) experiments with the collision gas (nitrogen) set to 5 psi (Khan et al., 2020). The de-clustering potential (DP) and collision energy (CE) for individual MRM transitions were obtained with further DP and CE optimization. The quantification of metabolites was accomplished using the targeted MRM approach (Khan et al., 2020). A specific set of MRM transitions were monitored for each period according to the metabolites within this period. Each sample analysis was conducted on both the positive and the negative modes. For the quality control (QC) of the metabolomic analysis, we pooled a QC sample by pipetting 10 μL from each sample, similar to the method used for assessing protein stability. One QC sample was analyzed after every 10 samples in the LC–MS/MS sequence. The stability of the LC–MS/MS analysis was evaluated by calculating the coefficient of variation (CV) of the peak area for each metabolite in the QC samples.

### 2.8 Metabolite identification and quantitation analysis

The MS data were processed using Software Analyst 1.6.3. The repeatability of metabolite extraction and detection was evaluated using the total ion current (TIC) and multiple peaks of MRM. Qualitative analysis of the first-order and second-order spectra detected by mass spectrometry was carried out on the basis of a home-made metadata database and existing metabolomic databases, including MassBank^1^ (Horai et al., 2010), HMDB^2^ (Wishart et al., 2018), LIPID MAPs^3^ (Sud et al., 2007) and Metlin^4^ (Xue et al., 2020).

Metabolites and therapeutic compounds with missing ratios exceeding 80% in a particular patient group were excluded from the metabolomics dataset. Only the metabolomics dataset containing endogenous metabolites was used for subsequent statistical analysis. Missing values in the dataset were imputed with the minimum value and zero. A two-sided unpaired Welch’s *t*-test was performed for each pair of comparing groups. Differential metabolites were further screened by combining the *p*-value and fold change from the univariate analysis. The screening criteria included metabolites with a fold change greater than or equal to 1.5 or less than or equal to 0.67, as well as a *p*-value less than 0.05. The pvalue were then adjusted using the Benjamini-Hochberg correction (qvalue <0.05).

Partial-least squares discrimination analysis (PLS-DA) (Ahn et al., 2020) was conducted as a supervised method to identify the important variables with discriminative power. PLS-DA models were validated based on the multiple correlation coefficient (R2), after that, we applied cross-validation on this R2 to calculate the cross-validated R2 (Q2); and permutation tests by applying 2000 iterations (*p* < 0.001).

### 2.9 Function enrichment analysis

We conducted GO and KEGG pathway enrichment analyses using the Gene Ontology (GO) database^5^ and Kyoto Encyclopedia of Genes and Genomes (KEGG) database^6^, respectively. These analyses aimed to explore the biological processes associated with the disease based on differential proteins and metabolites (Kanehisa et al., 2017; Zhou et al., 2019). The GO analysis included biological process (BP), cellular component (CC), and molecular function (MF) as the three main categories. R cluster Profiler (v3.12.0) package was used for enrichment analysis of differential molecules, pvalue was calculated by hypergeometric test, and pvalue was corrected by BH multiple hypothesis test. The adjusted *p*-values (FDR) < 0.05 was considered significant. Protein–protein interactions (PPI) between differential proteins were analyzed using metascape (Zhou et al., 2019), and networks were visualized with Cytoscape (v3.4.0) (Shannon et al., 2003).

In addition, for the pathway enrichment analysis of differential metabolome, we compared between groups according to the differences in metabolites, and used KEGG pathway and KEGG module for enrichment analysis. R cluster Profiler (v3.12.0) package with hypergeometric distribution test as pvalue <0.05 was used to determine significant enriched function that were enriched for at least three metabolites (for significantly altered metabolites), and fold enrichment >2.

### 2.10 Statistical analysis

Data analyses were performed using R software (version 4.1.2). The normality of the data distributions was checked using the Kolmogorov–Smirnov test. Normally distributed data are presented as the mean (±standard deviation). Principal component analysis (PCA) and hierarchical cluster analysis were performed using the distance matrix calculated using the pheatmap (Version 1.0.12, https://cran.r-project.org/web/packages/pheatmap/index.html), and ggord (Version 1.1.5). These statistical analyses were done with SAS, version 9.4 (SAS Institute, Inc., Cary, NC). Data visualization techniques utilized the ggplot2 package in R (version 4.1.2).

### 2.11 Data availability

The data that support the findings of this study will be available from the corresponding author upon reasonable request. The MS proteomics and metabolomics data have been deposited to the Proteome Exchange Consortium^7^ via the iProx partner repository (Ma et al., 2019) with the dataset identifier IPX0008710001.

## 3 Results

### 3.1 Proteomic and metabolomic profiling of patient’s plasma

The study included three cohorts matched for age and gender: a healthy control group (*n* = 18), recovered COVID-19 patients (*n* = 17), and long-term COVID-19 patients (*n* = 15). Table 1 shows baseline characteristics of patients enrolled in the study. At follow-up examination 6 months after discharge, we found that 15 patients with COVID-19 continued to exhibit at least a single clinical symptom (41%), including 13 severe patients (54%) and 9 non-severe patients (30%). The main symptoms among the patients were smell and taste dysfunction (27%), fatigue (27%), exertional dyspnea (33%), and muscular soreness (27%). Other symptoms included cough (20%), loss of appetite (20%) and nausea (7%).

**Table 1:** Demographics and clinical characteristics.

Characteristics	Healthy control (*n* = 18)	Recovered (*n* = 17)	Long COVID (*n* = 15)	*p* value
Sex – no. (%)				0.7819^a^
Male	10 (55%)	11 (57%)	8 (53%)	
Female	8 (45%)	6 (43%)	7 (47%)	
Age – year				
Mean ± SD	43 ± 4.78	42 ± 13.56	43 ± 13.22	0.2572^b^
Range	36–65	29–69	26–69	
Smoke – no. (%)		1 (6%)	2 (14%)	0.5887^c^
Alcohol – no. (%)		2 (12%)	1 (7%)	1^c^
Symptoms – no. (%)		3 (18%)	15 (100%)	1.47E-06^c^
Smell and taste dysfunction			4 (27%)	\
Fatigue			4 (27%)	\
Exertional dyspnea		1 (6%)	5 (33%)	\
Muscular soreness		1 (6%)	4 (27%)	\
Cough		1 (6%)	3 (20%)	\
Loss of appetite			3 (20%)	\
Nausea			1 (7%)	\

a Chi-square test.

b Student *t* test.

c Fisher exact test.

In our study, we collected plasma samples from subjects to conduct both proteomics and untargeted metabolomics analyses (Figure 1A). In summary, a total of 1,154 proteins were identified, and of these, 990 proteins were quantified and used for comparative analysis (Supplementary Table S1). And 1,082 metabolites were quantified through a compound library search. The coefficient of variation (CV) values of 93.2% of proteins were demonstrated to be <30% (Supplementary Figure S1A) in QC samples and meanwhile, the CV values of 90.3% metabolites were 30%, respectively (Supplementary Figure S1C). The median CVs for the proteomics and metabolomics data were 12.38 and 10.14%, respectively (Supplementary Figures S1B,D). These results indicate that the MS data were highly consistent and reproducible.

Further analysis using unsupervised PCA (Principal Component Analysis) was performed to investigate these components (Figures 1B,D). Our findings revealed significant differences in the proteomic profiles among the LC (Long COVID), Recovered (Recovered COVID-19), and HC (Healthy control) groups. The significant differences between groups of PROS, ZYX, TBA4A and ST1M1 genes may suggest that these genes can be used as important indicators of concern in LC group (Supplementary Figures S2A,B). Regarding the metabolomics analysis, the metabolic profiles of the LC and Recovered groups were similar to each other but differed significantly from the HC group. It is also mainly manifested by some differences in lipid metabolism (Supplementary Figures S2C,D). This suggests that both COVID-19 recovered patients and those in the LC group exhibit metabolic abnormalities. A pearson correlation coefficient test was used to analyze the sample-sample correlation among identified metabolites and proteins in healthy controls and COVID-19 patients. A heatmap was used to show the correlations between groups in the form of a matrix in Figures 1C,E. The results also showed that the overall metabonomics and proteomics profile of the LC group was different from that of the other two groups.

### 3.2 Characteristics of proteomics changes analysis

**Figure 2:**
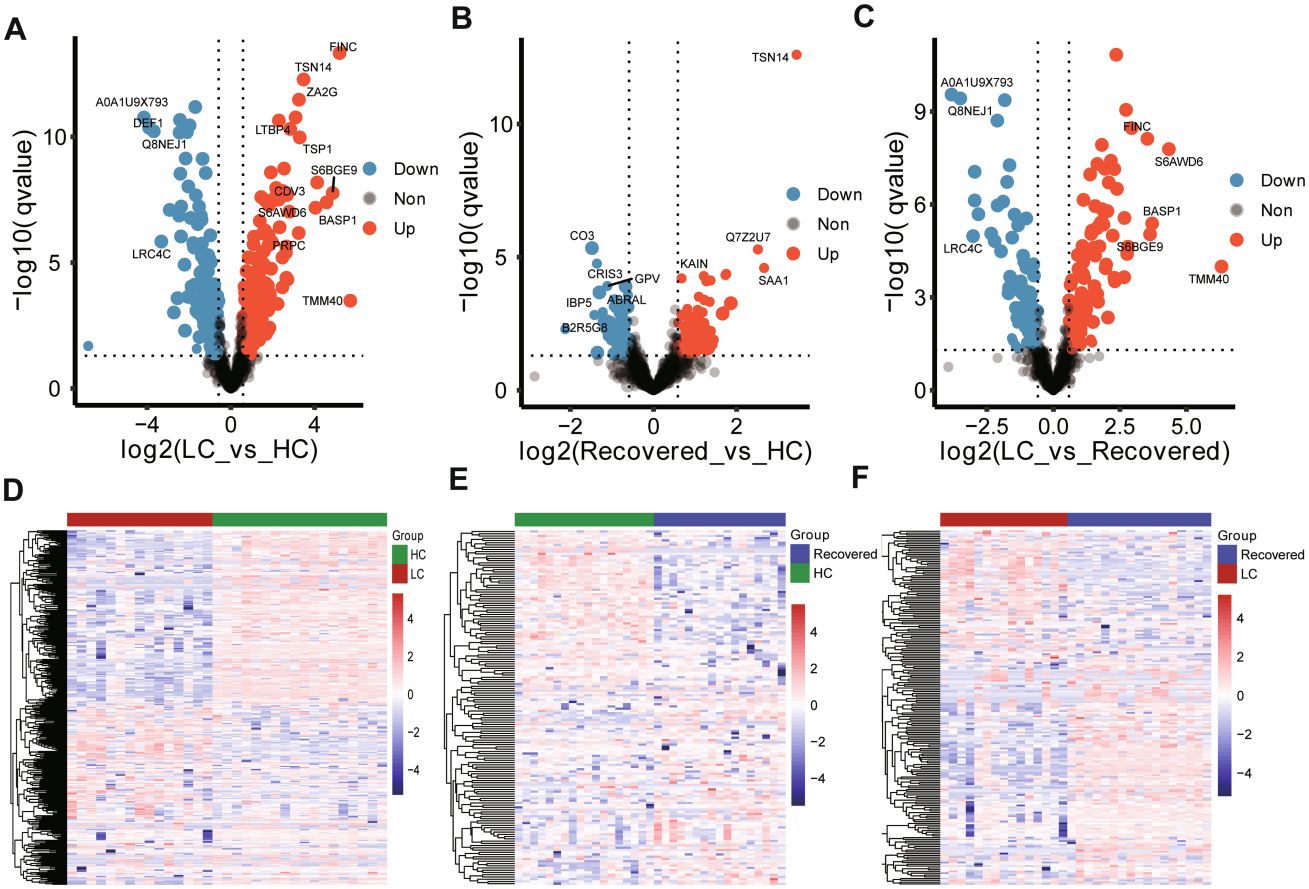
Expressed protein analysis of plasma samples from different group. Volcano plots show the change in transformed *p* value (−log10) against the log2 (Fold change) among in different compare group, LC-*vs*-HC **(A)**, Recovered-*vs*-HC **(B)** and LC-*vs*-Recovered **(C)**. Black dashed lines: cut-off values (log2|fold change| >0.67 (log2 (1.5) or and *p* value <0.05). Red dots: Highly detected protein in case group. Purple dots: Low expression of protein. Heatmap visualization of the significantly different expression proteins among in different compare group, LC-*vs*-HC **(D)**, Recovered-*vs*-HC **(E)** and LC-*vs*-Recovered **(F)**. DEPs included in the heatmap meet the requirement that fold change >1.5 or < 0.67 and *p* value (*t* test) of <0.05. Color bar represents the relative intensity of protein from −4 to 4.

Inter-group differential protein analysis was conducted using Welch’s *t*-test (Fold change >1.5 or Fold change <0.67, Pvalue <0.05 and qvalue with Benjamini-Hochberg less than 0.05). Compared to the HC group, the LC group had 187 proteins upregulated and 144 proteins downregulated (Supplementary Table S2). In comparison to the HC group, the Recovered group had 93 proteins upregulated and 65 proteins downregulated (Supplementary Table S3). When comparing the LC group to the Recovered group, 113 proteins were upregulated, and 86 proteins were downregulated (Supplementary Table S4). These findings are detailed in the volcano plots (Figures 2A–C) and heatmaps (Figures 2D–F). The results indicate a widespread variation in protein expression between the LC, Recovered, and HC groups.

### 3.3 Dysregulated immune response, coagulant functions and fatty acid transport from long COVID patients

**Figure 3:**
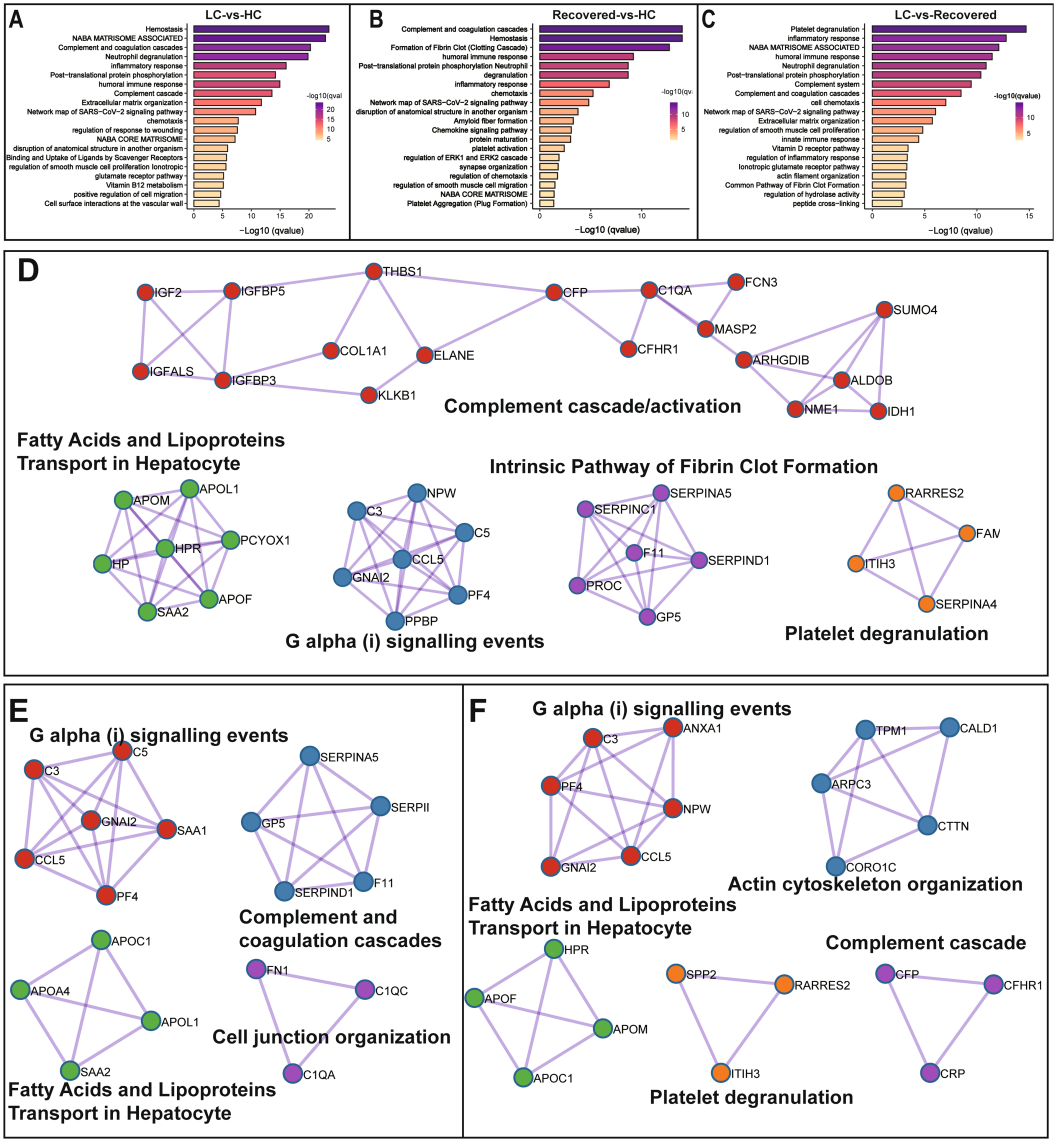
Differentially expressed protein enrichment analysis. Bar plot show the signaling pathway analysis of differential proteins among in different compare group, LC-*vs*-HC **(A)**, Recovered-*vs*-HC **(B)** and LC-*vs*-Recovered **(C)**. Functional enrichment analysis of PPI candidates among in different compare group, LC-*vs*-HC **(D)**, Recovered-*vs*-HC **(E)** and LC-*vs*-Recovered **(F)** *p* value is represented by color intensity.

We further performed pathway enrichment analysis on differentially expressed proteins, comparing with the HC group, pathways significantly enriched in the LC group included Hemostasis, Complement and coagulation cascades, and Neutrophil degranulation among other biological processes. Compared to the HC group, the Recovered group showed significant enrichment in biological processes such as Complement and coagulation cascades, Hemostasis, Formation of Fibrin Clot, humoral immune response, Post-translational protein phosphorylation, and Neutrophil degranulation. In comparison with the Recovered group, the primary biological processes significantly enriched in the LC group included Platelet degranulation, inflammatory response, humoral immune response, Neutrophil degranulation, and Post-translational protein phosphorylation (Figures 3A–C). The detail results of function enrichment analysis are shown in Supplementary Tables S5–S7. Moreover, based on known and predicted protein–protein interactions (PPI) databases, a PPI network analysis was conducted for differentially expressed proteins between groups. Compared to the HC group, the LC group displayed interactions among proteins involved in intracellular fatty acid and lipoprotein transport, the intrinsic pathway for fibrin clot formation, complement cascade/activation, G *α* (i) signaling events, and platelet degranulation, such as IGF2, IGFALS, CCL5, C1QA, KLKB1, etc. (Figure 3D). Similarly, compared to the HC group, the Recovered group’s PPI network interactions and nodes were relatively fewer, involving proteins in pathways similar to those in the LC group, such as G α (i) signaling events, complement and coagulation cascade reactions, cellular connectivity tissue, intracellular fatty acid, and lipoprotein transport (Figure 3E). Additionally, compared to the Recovered group, the LC group also showed dysregulation in pathways such as cytoskeleton organization, and fatty acid and lipoprotein transport (Figure 3F).

### 3.4 Characteristics of metabolomics changes analysis

**Figure 4:**
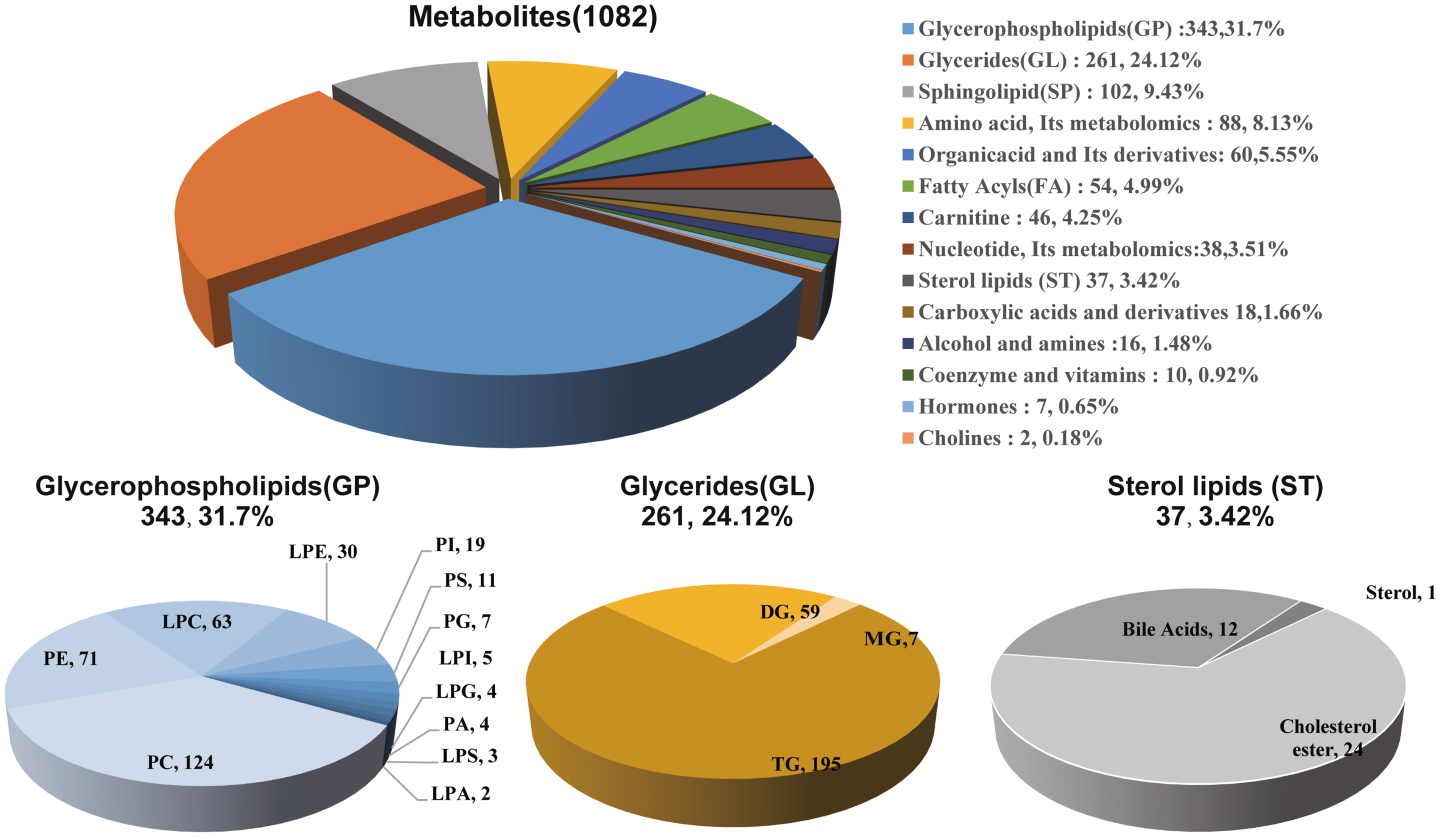
Composition analysis of plasma metabolites in patients. Among the 1,082 metabolites from all patients classified into 14 groups, a diverse assortment includes glycerophospholipids, glycerides, and sterol lipids, representing the major varieties associated with lipid within the classification.

First, we classified the 1,082 identified metabolites from all patients into 14 groups based on their product types (Supplementary Table S8). The major types included glycerophospholipids (31.7%), glycerides (24.12%), sphingolipids (9.43%), amino acids and their metabolites (8.13%), organic acids and their derivatives (5.55%), etc. Among them, glycerophospholipids (GP) were mainly composed of phosphatidylcholine (PC), phosphatidylethanolamine (PE), lyso-phosphatidylcholine (LPC), lyso-phosphatidylethanolamine (LPE), etc. Glycerides (GL) were mainly composed of triglyceride (TG), diglyceride (DG), etc. Additionally, lipid-related metabolites also included sterol lipids (3.42%), mainly consisting of 24 cholesterol species and 12 bile acids (Figure 4).

**Figure 5:**
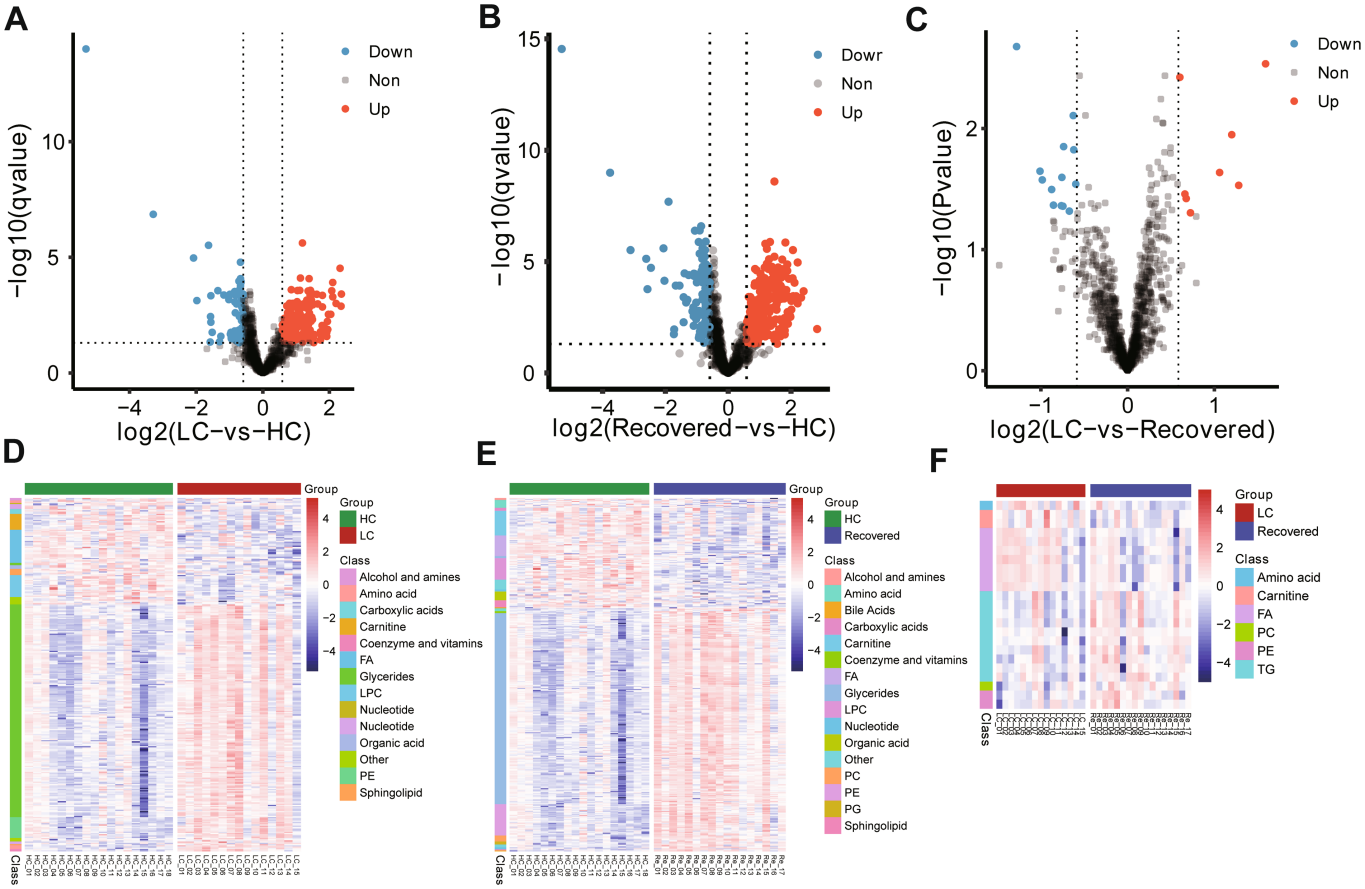
Expressed metabolite analysis of plasma samples from different group. Volcano plots show the change in transformed *p* value (−log10) against the log2 (Fold change) among in different compare group, LC-*vs*-HC **(A)**, Recovered-*vs*-HC **(B)** and LC-*vs*-Recovered **(C)**. Black dashed lines: cut-off values (log2|fold change| >0.67 (log2(1.5) or and *p* value <0.05). Red dots: Highly detected metabolites in case group. Purple dots: Low expression of metabolites. Heatmap visualization of the significantly different altered metabolites among in different compare group, LC-*vs*-HC **(D)**, Recovered-*vs*-HC **(E)** and LC-*vs*-Recovered **(F)**. DEPs included in the heatmap meet the requirement that fold change >1.5 or < 0.67 and *p* value (*t* test) of <0.05. Color bar represents the relative intensity of altered metabolites from −4 to 4.

Using the Welch *t*-test for inter-group difference analyses of metabolites (Fold change >1.5 or Fold change <0.67, Pvalue<0.05 and qvalue with Benjamini-Hochberg less than 0.05), in comparison to the HC group, the LC group showed upregulation in 180 metabolites, such as glycerides (primarily triglycerides), and PE, while 81 metabolites were downregulated, notably including choline, fatty acids (especially long-chain fatty acids), and lyso-phosphatidylcholine (Figures 5A,D). Compared to the HC group, the Recovered group exhibited upregulation in 241 metabolites, such as triglycerides, PE, and phosphatidylcholine. Conversely, 112 metabolites were downregulated, including choline, long-chain fatty acids, and lyso-phosphatidylcholine (Figures 5B,E). Interestingly, according to the definition criteria of differential metabolites, the LC group and the RE group did not have any different metabolites. Filtering with fold change >1.5 or Fold change <0.67 and pvalue <0.05 was different for only 22 metabolites, including upregulation in 10 metabolites, such as amino acids. And downregulation was observed in 12 metabolites, including triglycerides (Figures 5C,F). These results suggest that the specific metabolites in the Recovered group and the LC group were not significantly different. The detail result of differentially altered metabolites are shown in Supplementary Tables S9–S11.

Furthermore, by employing partial-least squares discrimination analysis (PLS-DA) for supervised analysis, we distinguished between samples from different groups (Supplementary Figure S3). The results showed a clear classification between the LC and HC groups, with R2Y and Q2Y values of 0.964 and 0.811, respectively, indicating significant metabolic spectrum differences between the two groups (Supplementary Figure S3A). Similarly, a clear classification between the Recovered and HC groups was observed, with R2Y and Q2Y values of 0.992 and 0.773, respectively, suggesting significant metabolic spectrum differences between these groups as well (Supplementary Figure S3B). However, due to the smaller overall differences between the LC and Recovered groups, PLS-DA analysis was unable to construct a model, indicating minor overall metabolic spectrum differences between LC and Recovered.

### 3.5 Dysregulated lipid metabolism from long COVID and COVID-19 recovered patients

**Figure 6:**
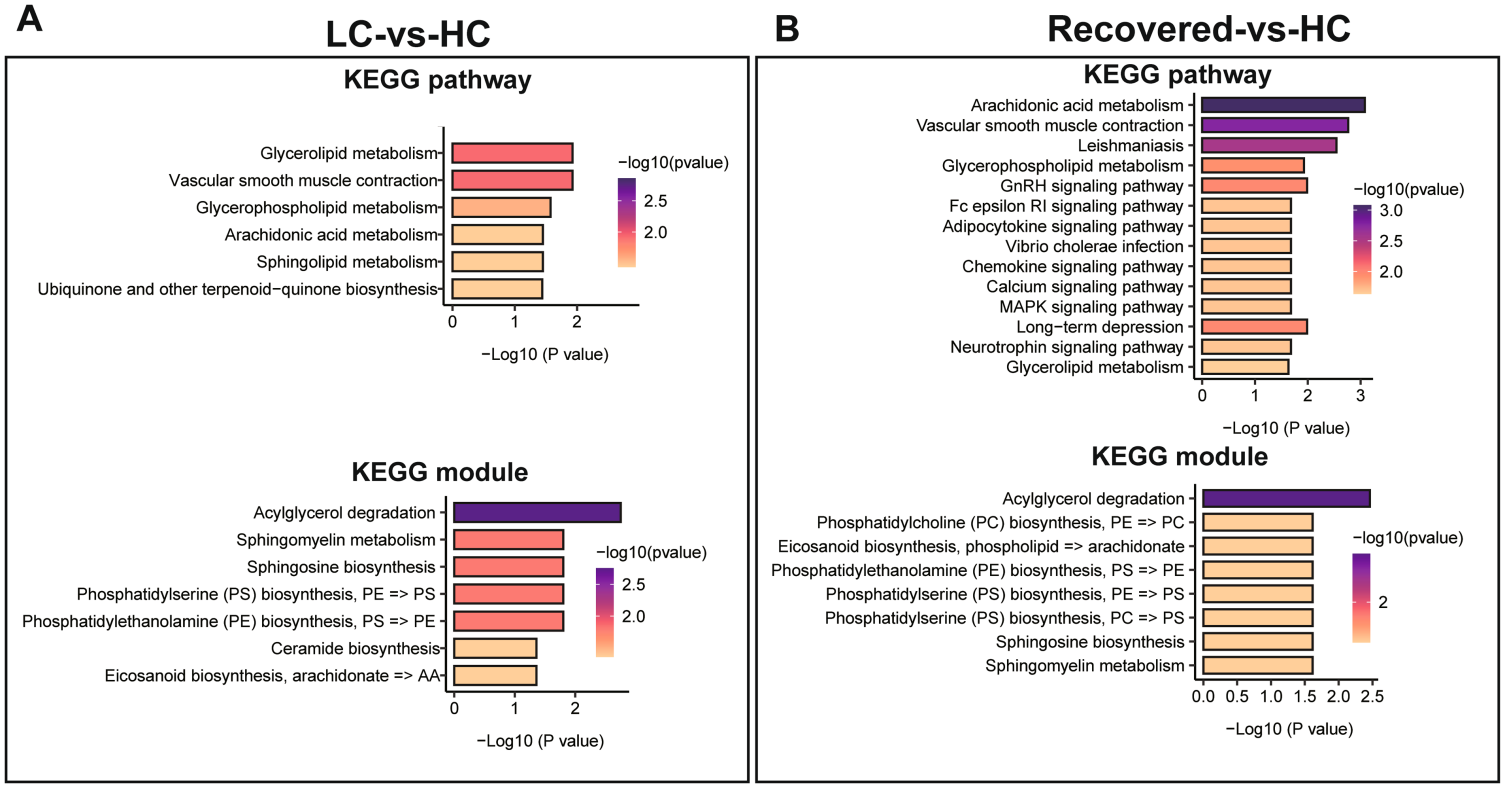
Differential metabolite of KEGG enrichment analysis. **(A)** KEGG pathways enrichment analysis and KEGG module analysis between LC and HC group. **(B)** KEGG pathways enrichment analysis and KEGG module analysis between Recovered and HC group. *p* value is represented by color intensity.

We further analyzed differential metabolites among groups through KEGG pathway enrichment analysis (Supplementary Tables S12, S13) and KEGG module analysis to investigate the potential functional mechanisms and biological significance of specific pathways and units. Compared to the HC group, significant enrichment in lipid-related metabolic pathways was observed in the LC group, including Glycerolipid metabolism, Glycerophospholipid metabolism, Sphingolipid metabolism, among others, as well as metabolic pathways such as Arachidonic acid metabolism and Vascular smooth muscle contraction. The functional units involved mainly pertain to the biosynthesis or degradation of lipid substances, like the synthesis of Sphingosine, Phosphatidylserine (PS), Phosphatidylethanolamine (PE), Ceramide, and the degradation of Acylglycerol. Additionally, fatty acid biosynthesis processes such as Eicosanoid biosynthesis are included (Figure 6A).

Compared to the HC group, the pathways significantly enriched in the Recovered group, besides lipid metabolism pathways like Glycerophospholipid metabolism and Glycerolipid metabolism, include endocrine signaling pathways such as the GnRH signaling pathway, immune-related signaling pathways like the Fc epsilon RI signaling pathway and the Chemokine signaling pathway, and pathways regulating cellular functions such as the MAPK signaling pathway and the Calcium signaling pathway. The corresponding functional units, similar to those in the LC group, mainly involve the biosynthesis or degradation of lipid substances and fatty acids, including PC biosynthesis, Eicosanoid biosynthesis, Acylglycerol degradation, among others (Figure 6B).

## 4 Discussion

Long COVID is a multisystem disease, often presenting as severe symptoms following infection with Severe Acute Respiratory Syndrome Coronavirus 2 (SARS-CoV-2), including but not limited to myalgia, breathing difficulties, abnormalities in chest imaging and pulmonary function tests, and cardiovascular problems (Carfì et al., 2020; Davis et al., 2021; Daitch et al., 2022). However, there are no uniform guidelines for LC-19 to assist in standardized and earlier detection; diagnosis is typically made by assessing symptoms and ruling out other diseases (Srikanth et al., 2023). Thus, exploring the pathophysiological mechanisms of Long COVID is crucial for identifying therapeutic targets for the disease, enabling the establishment of appropriate treatment and clinical management strategies for early diagnosis. This study included Long COVID patients, COVID-19 recovered patients, and healthy control subjects, investigating the molecular characteristics and potential pathogenic mechanisms of Long COVID patients through proteomic and metabolomic analyses of plasma samples.

Our research findings indicate that, compared to healthy subjects, protein dysregulation in Long COVID patients primarily involves pathways like coagulation, platelets, complement cascade reactions, GPCR cell signal transduction, and substance transport, which participate in regulating immune responses, inflammation, and tissue repair. Abnormalities in the coagulation system (fibrin clot, platelet activation, coagulation cascade reaction), especially among severe COVID-19 patients, have been shown to be associated with disease progression and may lead to severe complications (Al-Samkari et al., 2020) such as deep vein thrombosis (DVT), disseminated intravascular coagulation (DIC). Furthermore, coagulation system abnormalities are involved in thrombus formation and can release pro-inflammatory and pro-coagulant factors, further enhancing inflammation and coagulation. Additionally, the complement cascade reaction is a key pathway for immune defense, with abnormalities causing immune dysregulation (Warwick et al., 2021). Complement activation products like C3a and C5a exert their effects through their corresponding GPCRs, which are critical for regulating inflammatory responses and the recruitment of immune cells (Wang et al., 2019). Similar to our study, recent research found disrupted plasma proteomes within 12 weeks post-COVID-19 recovery, characterized by the differential expression of proteins involved in lipid metabolism, complement, and coagulation cascades (Captur et al., 2022). Clinical studies have also observed immune and coagulation dysfunctions in patients, identifying persistent coagulopathy and elevated antifibrinolytic levels in Long COVID patients (Pretorius et al., 2021). In some patients, a pro-coagulant state, endothelial dysfunction, and inflammation can be detected about a year post-COVID-19 recovery (Fan et al., 2022). Additionally, compared to COVID-19 recovered patients, Long COVID patients exhibit significant proteomic differences, exhibiting more pronounced dysregulation of coagulation, platelet, and immune-related proteins, as well as disturbances in cytoskeleton, lipoprotein pathways, further supporting sustained chronic inflammation and vascular dysfunction in Long COVID patients (Ong et al., 2021).

Proteins primarily related to immune system, coagulation system, and signal transduction system dysregulation in this study include: Insulin-like growth factor-related proteins (IGF-2, IGFALS, IGFBP3), inflammation-related proteins (C1QA, MASP2, CFHR1, SAA2), coagulation-related proteins (KLKB1, ELANE, PROC, SEPRINC1, SERPIND1, F11), G protein signal transduction-related proteins (C3, C5, CCL5, GNAI2, PF4), lipid transport (APOL1, APOM, APOF), among others, with multiple proteins interacting and regulating homeostasis within the body. Previous studies have associated IGFBP-2, IGF1 with adverse outcomes in COVID-19 patients (Ilias et al., 2021; Mester et al., 2024). Liew found that increased complement activation marker C1QA is associated with the long presence of the coronavirus (Liew et al., 2024). Carlo also discovered complement dysregulation accompanied by inflammatory signatures in active Long COVID patients (Cervia-Hasler et al., 2024). Lower respiratory tract levels of CCL5 are associated with high viral loads of SARS-CoV-2 (Pérez-García et al., 2022), and with inflammation, atherosclerosis complications of COVID-19 (Das and Podder, 2021). Meta-analyses have indicated that GPCRs may play a significant role in Long COVID symptoms (Akbari et al., 2023) (Supplementary Figure S2). Moreover, researchers using machine learning discovered that CXCL5, AP3S2, MAX, PDLIM7, and FRZB could differentiate Long COVID outpatients from healthy control subjects, with protein functions related to immune cell activation, and nervous system development (Patel et al., 2023). Therefore, our study’s results include key proteins associated with Long COVID dysregulation identified in previous research, as well as other potential proteins closely related to Long COVID symptoms. For instance, acute kidney damage caused by COVID-19 may lead to tubular damage, endothelial damage, and glomerular injury (LONG et al., 2022), with APOL1, APOM, APOF possibly being related to glomerular diseases in Long COVID symptoms. KLKB1, serine protease inhibitors, among others, are associated with atherosclerosis and the coagulation cascade (Moellmer et al., 2024; Wang et al., 2022; Rein et al., 2011). The critical mechanisms of these potential proteins still require further investigation.

In addition to proteomics, this study further examined the metabolite levels in the plasma of subjects. Compared with healthy subjects, both Long COVID patients and COVID-19 recovered patients exhibited similar metabolic disorders, primarily involving dysfunctions in lipid metabolites and fatty acid metabolism, such as glycerophospholipids, sphingolipid metabolism, and arachidonic acid metabolism. These involve biological processes such as phosphatidylserine, sphingomyelin and ceramide biosynthesis, and acylglycerol degradation, indicating that Long COVID still features impaired energy metabolism, chronic immune dysregulation, and dyslipidemia. Glycerophospholipids, as major components of cellular membranes, participate in processes like signal transduction, energy storage, inflammation, apoptosis, and lipid transport (Sakuragi and Nagata, 2023). Plasma levels of glycerophospholipids, including phosphatidic acids and phosphatidylinositol, are reduced in COVID-19 patients (Song et al., 2020) (Supplementary Figure S3). Sphingolipids, key bioactive molecules involved in inflammation, cell differentiation, regeneration, and particularly important in musculoskeletal cells, have their metabolic dysregulation possibly related to fatigue and muscle pain (Meacci et al., 2022). Researchers found that, compared with the control group, the expression levels of phosphatidylcholine and sphingomyelin were higher in the blood of patients 2 years after COVID-19 infection (López-Hernández et al., 2023). Ceramides, derivatives of sphingomyelin, have been found to increase expression in patients with chronic fatigue syndrome (Filippatou et al., 2021). Arachidonic acid is a precursor to pro-inflammatory prostaglandin E2 and anti-inflammatory lipoxin A4, making arachidonic acid metabolism crucial in the activation and resolution of inflammation (Das, 2021). Metabolites of arachidonic acid can serve as potential biomarkers for COVID-19 (Ghimire et al., 2023). Furthermore, in addition to the dysregulated expression of lactate and pyruvate, patients with Long COVID syndrome also exhibit disorders in lipid metabolites, such as triglycerides, apolipoproteins, and long-chain acylcarnitine (Berezhnoy et al., 2023; Yang et al., 2022). Thus, similar to previous metabolomic studies, the comorbidities of Long COVID may be due to prolonged dysregulation of inflammatory metabolites and lipid metabolites.

Despite the progress and findings, this study has several limitations, highlighting the necessity and direction for future research. Firstly, a major limitation is the small sample size of the cohort. As the number of participants was limited, the results of this study may be subject to a certain degree of bias. The small sample size limits our ability to generalize the findings to a broader population and may lead to statistical uncertainties. Secondly, there is a lack of clinical biochemical markers data in this study. Biochemical markers could provide deeper insights into physiological mechanisms, and their absence limits our understanding of the complex mechanisms behind the study subject. In future research, collecting and analyzing relevant biochemical markers will be key to understanding these processes, thereby aiding in revealing the disease’s pathogenesis or evaluating the effectiveness of health interventions.

## 5 Conclusion

In conclusion, this study rigorously explored the persistent changes in protein and metabolite levels in the plasma of Long COVID-19 patients, revealing significant biomarker abnormalities within these individuals compared to recovered patients. Through comparative analysis, our findings clearly demonstrate significant protein dysregulation and metabolic abnormalities in the plasma of Long COVID-19 patients, more pronounced than in those who have recovered from COVID-19. Our results provide a metabolic and proteomic molecular profile of Long COVID-19 patients, mapping out specific changes in proteins and metabolites in their plasma. This not only aids in the exploration of biomarkers but is also crucial for understanding the complex pathogenesis and pathophysiological foundation of Long COVID-19.

## Data Availability

The datasets presented in this study can be found in online repositories. The names of the repository/repositories and accession number(s) can be found in the article/Supplementary material.

## Footnotes

^1^ http://www.massbank.jp/

^2^ http://www.hmdb.ca/

^3^ www.lipidmaps.org/data/structure/

^4^ http://metlin.scripps.edu/index.php

^5^ http://geneontology.org/

^6^ http://www.genome.jp/kegg/

^7^ http://proteomecentral.proteomexchange.org
