# Supplementary material for: Proteomic and metabolomic profiling of plasma uncovers immune responses in patients with Long COVID-19
Source: Front Microbiol. 2024 Dec 27;15:1470193. doi: 10.3389/fmicb.2024.1470193 (PMC11718655; doi:10.3389/fmicb.2024.1470193)
Supplement: Supplementary file 1 [file Data_Sheet_1.pdf]

Supplementary Material

Supplementary Figures

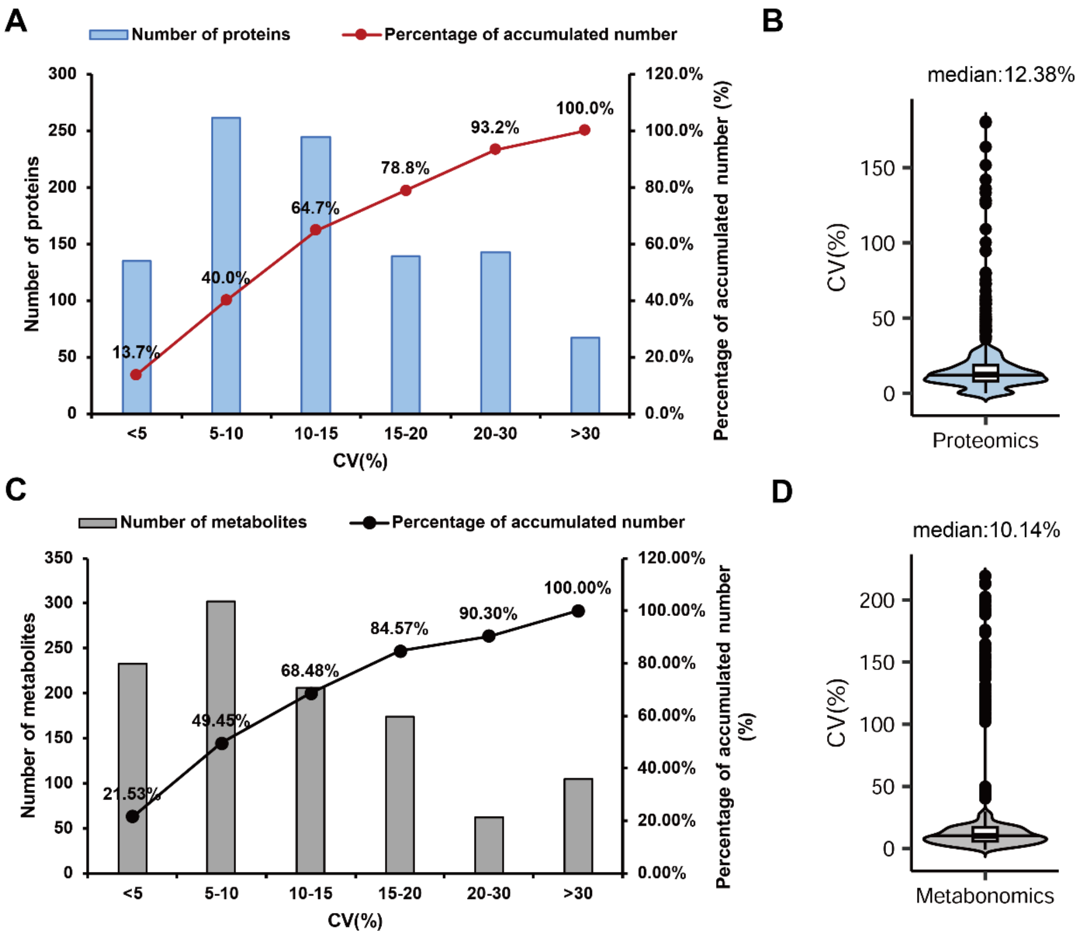

Supplementary Figure 1. Quality Control of Proteomics and Metabolomics Data.

CV distribution plot(A) and boxplot (D) of QC samples in proteomics data.

CV distribution plot(C) and boxplot (D) of QC samples in metabonmics data.

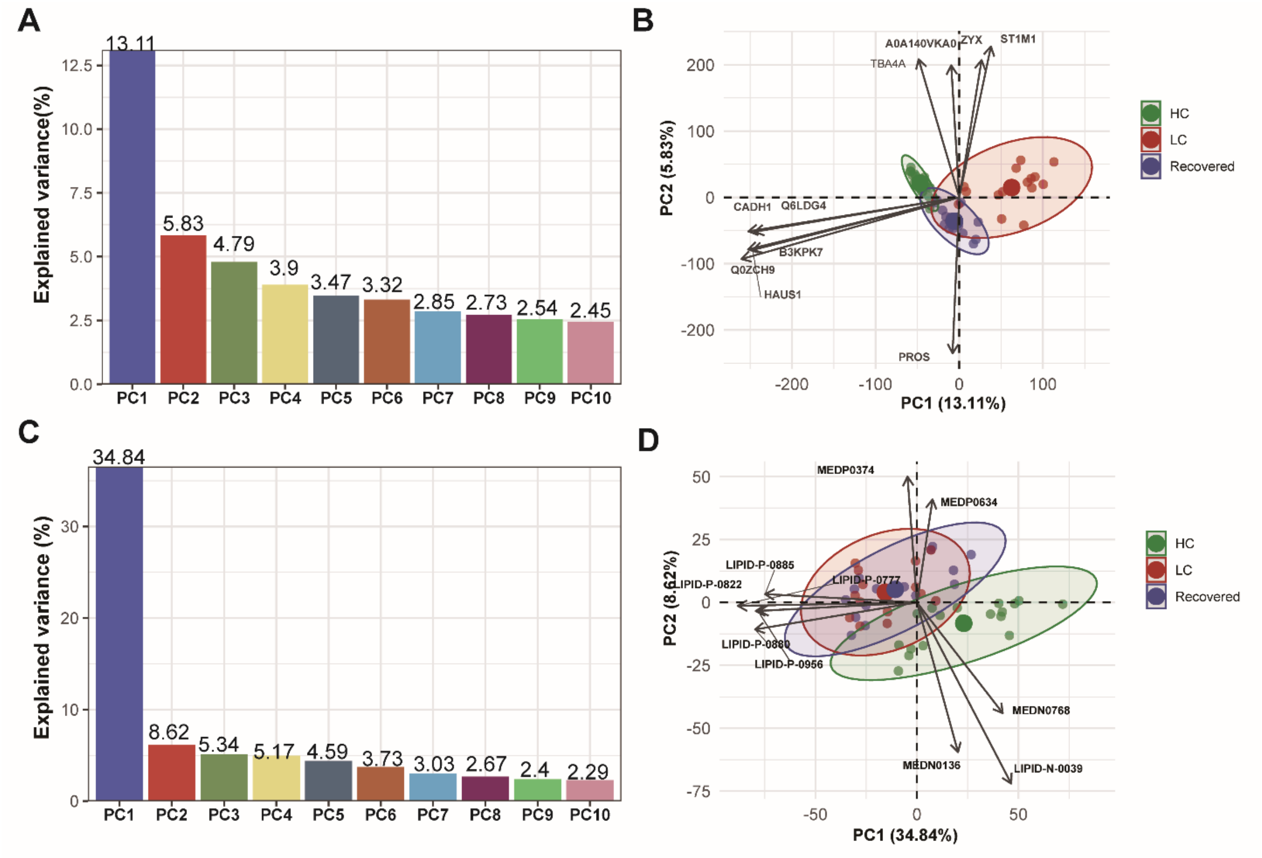

Supplementary Figure 2. PCA analysis of Proteomics data and metabolomics data.

Principal component lithotripsy diagram from proteomics data(A) and metabonomics data (C), the plot of PCA weights from proteomics data (B) and metabonomics data (D).

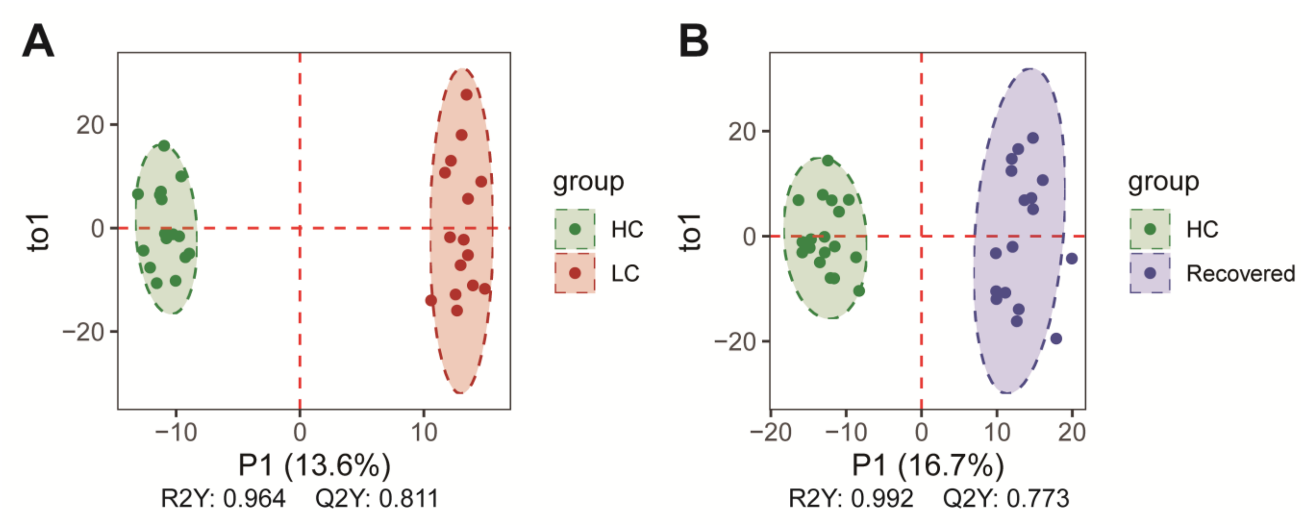

Supplementary Figure 3. PLS-DA analysis results. LC-vs-HC(A), Recovered-vs- HC(B)

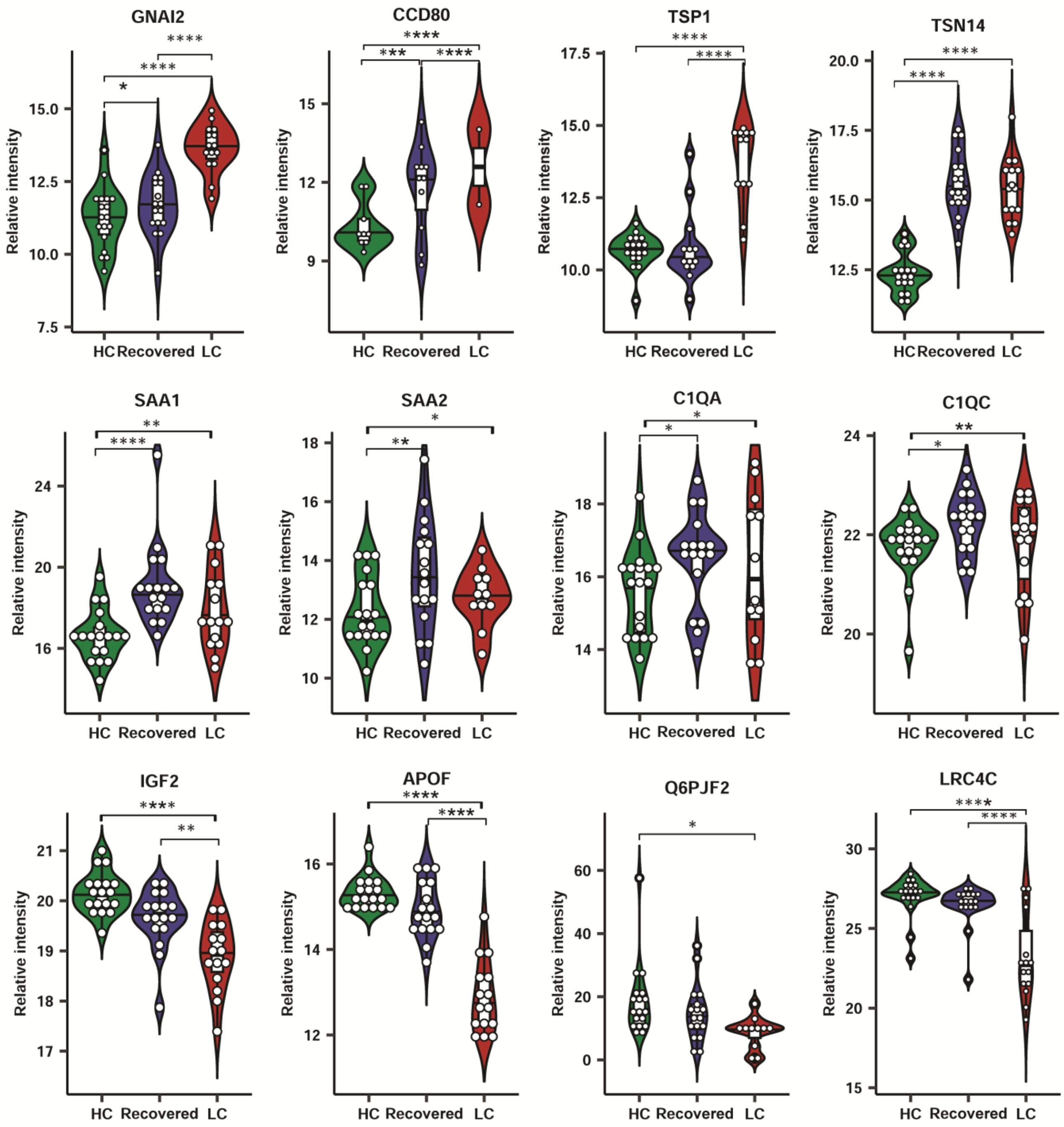

Supplementary Figure 4. Relative abundance of differentially expressed proteins (DEPs).

∗ p-value <0.05 ∗∗, p-value <0.01; ∗∗∗, p-value <0.001; ∗∗∗∗, p-value <0.0001.

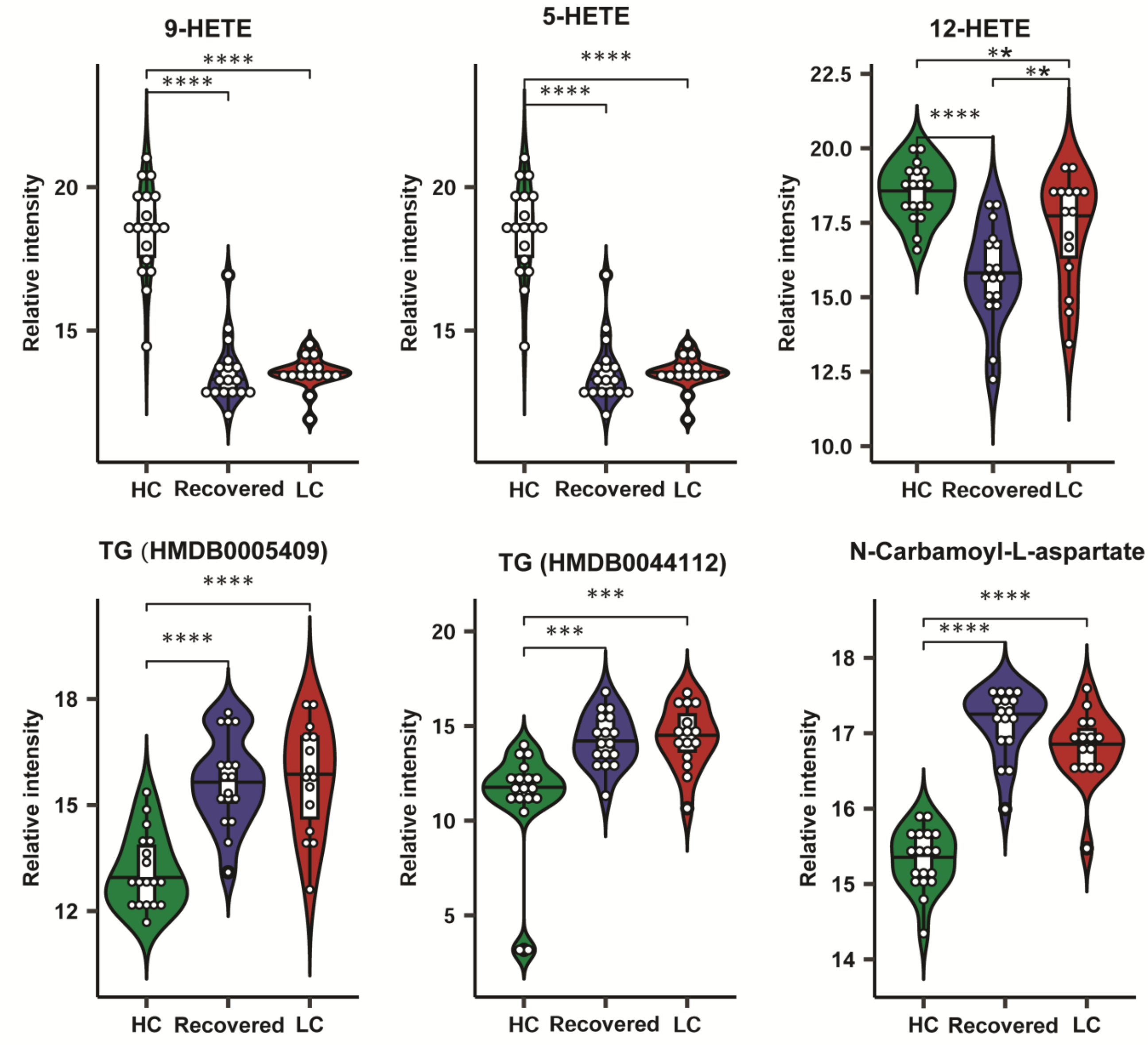

Supplementary Figure 5. Relative abundance of key metabolites.

∗ p-value <0.05 ∗∗, p-value <0.01; ∗∗∗, p-value <0.001; ∗∗∗∗, p-value <0.0001.
